# Genital Infection Caused by *Salmonella enterica* Serovar Hvittingfoss: A Case Report

**DOI:** 10.3390/pathogens12111316

**Published:** 2023-11-05

**Authors:** Emilie De Hert, Sarah Baïli, Marleen Vanden Driessche, Hilde Jansens, Sarah Vandamme, Yves Jacquemyn, Alexandra Vodolazkaia, Marina Mukovnikova, Wesley Mattheus, Veerle Matheeussen

**Affiliations:** 1 Department of Microbiology, University Hospital Antwerp, 2650 Edegem, Belgiumveerle.matheeussen@uza.be (V.M.); 2 Department of Obstetrics and Gynecology, University Hospital Antwerp, 2650 Edegem, Belgium; 3 National Reference Centre for Salmonella and Shigella Species, Sciensano, 1050 Brussels, Belgiumwesley.mattheus@sciensano.be (W.M.); 4 Laboratory of Medical Microbiology, Vaccine & Infectious Disease Institute (VAXINFECTIO), University of Antwerp, 2610 Wilrijk, Belgium; 5 Laboratory of Medical Biochemistry, University of Antwerp, 2610 Wilrijk, Belgium

**Keywords:** *Salmonella enterica* serovar Hvittingfoss, endometritis, bacteremia

## Abstract

Background: Nontyphoidal *Salmonella* serovars predominantly cause gastrointestinal infections. However, other clinical presentations, including urogenital infections, have been reported, although they are rather rare. Case presentation: This case is about a 33-year-old woman diagnosed with *Salmonella enterica* serovar Hvittingfoss (*S.* Hvittingfoss) bacteremia and endometritis six days post uterine aspiration in the context of a missed abortion. She had traveled to Indonesia two weeks prior to the positive blood and cervical culture. She never developed gastrointestinal symptoms but was found to carry *S.* Hvittingfoss in her stool sample. The patient was successfully treated with a seven-day course of iv ciprofloxacin. Conclusions: *S.* Hvittingfoss is a rare serovar that has caused a few outbreaks of foodborne infections in Asia, the United States, and Australia. To the best of our knowledge, this is the first reported case of *Salmonella* urogenital infection caused by this serovar. *Salmonella* as a cause of urogenital infections is rare but not uncommon. Therefore, it should be considered in identifying members of the *Enterobacterales* among urogenital flora in cases of severe urogenital infections, especially when other cultures remain negative.

## 1. Introduction

*Salmonellae*, Gram-negative motile bacilli belonging to the *Enterobacterales* family, cause mostly foodborne gastrointestinal infections. Salmonellosis frequently presents as diarrhea associated with fever and abdominal cramping. In most cases, the infection resolves spontaneously in 4–7 days, but bacteremia or focal infections can occur [1]. Atypical presentations of salmonellosis, including urogenital infections [2,3,4], have been reported, although they are rather rare. Here, we report a case of a genital infection with bacteremia caused by *Salmonella enterica* serovar Hvittingfoss (*S.* Hvittingfoss), a serovar rarely observed in Belgium.

## 2. Case Description

Two sets of blood culture collected via venipuncture in BacT/Alert^®^ FA Plus and FN Plus bottles (bioMérieux, Schaarbeek, Belgium) from a 33-year-old woman who presented at the emergency unit with fever and lower abdominal pain showed aerobic and anaerobic growth after approximately 12 h of incubation. Direct microscopic examination and Gram staining showed mobile, Gram-negative rods, which were identified as *Salmonella* species with matrix-assisted laser desorption/ionization time of flight (MALDI-TOF) MS (Bruker, Kontich, Belgium). The slide agglutination test (Sifin, Berlin, Germany) showed the presence of a nontyphoidal *Salmonella*. The patient was admitted to the hospital on suspicion of endometritis six days post uterine aspiration in the context of a missed abortion at six weeks gestational age. Culture of a cervical swab only showed moderate growth of normal urogenital flora, including *Enterobacterales*. The missed abortion was diagnosed during a 6-week trip in Indonesia, 5 weeks prior to the uterine aspiration. Misoprostol was used during that trip, but unsuccessfully.

In search of the focus of the *Salmonella* bacteremia and to check for possible asymptomatic *Salmonella* carriage, urine and stool samples were requested. The cervical sample was reexamined specifically for *Salmonella* species. The urine culture was negative, but the cervical culture showed moderate growth of *Salmonella* species, and the stool culture was positive for *Salmonella* species. The patient did not experience gastrointestinal symptoms during her trip to Indonesia, but her partner did. However, she experienced abdominal cramps and diarrhea, which could be attributed to the use of misoprostol for medical treatment of a missed abortion. Unfortunately, the causes of the gastrointestinal symptoms of the partner were never investigated.

**Figure 1:**
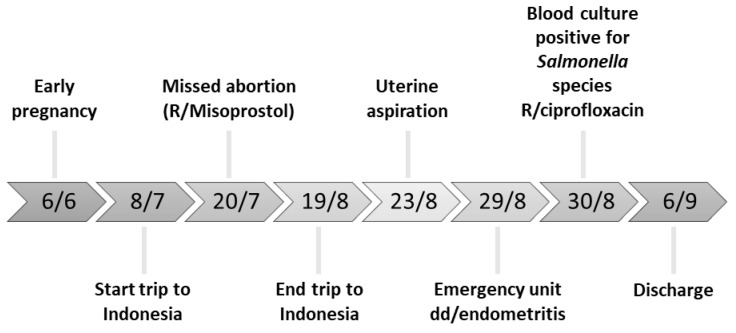
Time course of the described clinical case.

Antibiotic sensitivity testing via disk diffusion showed that the *Salmonella* strain was sensitive to ciprofloxacin, amoxicillin-clavulanic acid, piperacillin-tazobactam, cefuroxime, ceftriaxone, ceftazidime, cefepime, meropenem, amikacin, and co-trimoxazole. Empiric antibacterial therapy (900 mg Clindamycin/8u iv and 90 mg gentamicin/8u iv, due to penicillin allergy) was switched to 400 mg ciprofloxacin iv, twice a day. After 7 days of ciprofloxacin iv therapy, the patient was discharged in good clinical condition. The follow-up appointment 3 weeks later was reassuring (see Figure 1 for the time course of the case).

**Figure 2:**
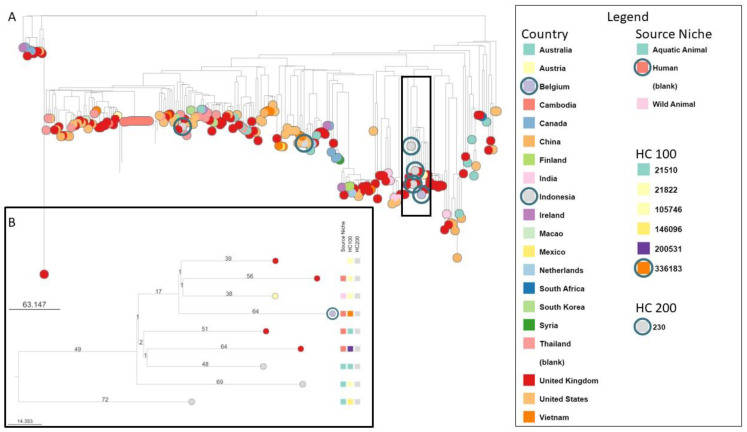
(**A**) Comparison of *S*. Hvittingfoss strains found in Enterobase carried out with cgMLST v2 hierarchical clustering group HC400 = 230 (*N* = 227, assessed 23 November 2022). This case’s isolated strain (purple dot and encircled) showed closest similarity with a few strains from the UK (*N* = 4), Indonesia (*N* = 3) and Austria (*N* = 1). Given the recent travel history of the patient, strains originating from Indonesia are also encircled (gray dots) and tend to concentrate in the same branch. (**B**) Subtree of most related strains found in Enterobase. Minimal spanning tree created with Grapetree using MSTree V2 with default settings and visualized with Microreact.

The three *Salmonella* isolates (cervix, blood, and stool) were sent to the Belgian *Salmonella* Reference Center (Sciensano) for further serotyping. All three isolates were identified as *Salmonella enterica* serovar Hvittingfoss by slide agglutination. Whole Genome Sequencing (WGS) proved that the three isolates were identical. Comparison with isolates available in the international database Enterobase (assessed 23 November 2022) showed similarity with strains from the UK (*N* = 4), Indonesia (*N* = 3), and Austria (*N* = 1) (Figure 2). 

## 3. Discussion

Although *Salmonella* species predominantly cause gastrointestinal infections, other clinical presentations have been reported. Here, we report an atypical presentation of salmonellosis caused by a rare serovar: *Salmonella* endometritis and bacteremia caused by *S.* Hvittingfoss. 

Although *Salmonella* is not a typical causative agent of urogenital infections, different nontyphoidal *Salmonellae* (NTS) have been reported as causes of pelvic abscesses [2,3,4]. One case report described a pelvic inflammatory disease caused by *Salmonella* O serogroup C2 that was not preceded by gastrointestinal symptoms. The patient responded well to abscess drainage and was discharged with four weeks of ciprofloxacin treatment. However, she had a relapse of the pelvic symptoms, and eventually a hysterectomy was required for full recovery [2]. Another case report addressed acute salpingitis, whereby *Salmonella* Panama was isolated from peritoneal pus as well as from stool samples. The patient recovered after 14 days of ciprofloxacin treatment, but recurrence eventually required the removal of the uterus, fallopian tubes, and the right ovary [3]. A third case report informed us about pelviperitonitis, accompanied by acute sepsis caused by *Salmonella* Reading. The patient fully recovered after abscess drainage and doxycycline, metronidazole, and ceftriaxone administration. The woman did not experience diarrhea before symptom onset [4]. Also, a *Salmonella* bacteremia after confirmed postpartum endometritis has been described before. The patient fully recovered after antibacterial therapy [5]. These cases prove that urogenital infections caused by *Salmonella* can have distinct clinical manifestations with different degrees of severity or complications, sometimes necessitating invasive surgery accompanied by antibacterial therapy. 

Interestingly, the woman described in our case did not experience gastrointestinal symptoms, except for abdominal cramps and diarrhea after taking misoprostol, before the development of the *Salmonella* bacteremia and endometritis, which is similar to two of the three *Salmonella* urogenital infections described above [2,3,4], indicating asymptomatic *Salmonella* carriage. The occurrence of NTS asymptomatic carriers in the general population is rather unknown, as long-term NTS infections are less studied than typhoidal *Salmonella* infections. In 2019, a review on persistent infections and long-term carriages of NTS concluded that more than 2% of patients infected with NTS become temporary carriers for 1–12 months. Asymptomatic carriage for years is rarely described, and lifelong carriage was never detected [6]. *Salmonella* carriage in adults who have never experienced gastroenteritis before is understudied because, in most cases, it remains undetected. A study with 3000 asymptomatic hotel workers from Bangkok suggested that long-lasting NTS asymptomatic carriage is rare, but nevertheless, 4.7% of the healthy participants were asymptomatic NTS carriers [7]. 

In our case, the first microbiological examination of the cervical culture was reported to be negative for urogenital pathogens; only moderate growth of urogenital flora was reported. However, a reexamination with a specific focus on the detection of *Enterobacterales* revealed the presence of *Salmonella* species. This is, however, not part of the routine urogenital culture protocol, which is in line with The Clinical Microbiology Procedures Handbook, which states that *Enterobacterales* are part of the colonizing flora of the female genital tract. Identification of enteric pathogens is only necessary when the specimen is invasively collected, when there is heavy growth of these pathogens, or when the pathogens are the predominant microorganisms in the culture [8]. In our urogenital culture protocol, the following culture plates are inoculated with the cervical swab: a blood agar plate for aerobic growth, a Columbia Nalidixic Acid agar plate with human blood, and a Sabouraud Glucose Selective agar plate. A blood agar plate for anaerobic growth is only inoculated upon request of the clinician. On the inoculated agar plates, *Salmonellae* cannot be distinguished from other *Enterobacterales* and was therefore first reported as urogenital flora. 

Interestingly, the hypothesis on the role of *Salmonella* in this case is difficult to make unambiguously: was the missed abortion a consequence of the *Salmonella* infection or did the *Salmonella* infection spread to the endometrium and the circulatory system due to the uterine aspiration? Although extremely rare, transplacental passage of *Salmonella* leading to miscarriage or stillbirth has been described [9,10,11]. A literature search revealed no cases of *Salmonella* pelvic infection following uterine aspiration. It is very likely that the *Salmonella* infection was contracted in Indonesia, given the similarity of the *S.* Hvittingfoss strain with Indonesian strains in the international database. Moreover, the missed abortion did occur during that trip. On the other hand, the patient did only show symptoms of infection five days post uterine aspiration, and she did not receive antibiotic prophylaxis prior to the procedure. It is nevertheless recommended by the Belgian Society of Infectiology and Microbiology to give antibiotic prophylaxis prior to uterine aspiration in first-trimester pregnancies in the form of cefazolin or cefuroxime, or aztreonam in cases of penicillin allergy. This information points in the direction of the uterine aspiration as the cause of the *Salmonella* endometritis and bacteremia. 

Serotyping identified *S.* Hvittingfoss as the cause of this infection. This serovar is rarely observed in Belgium (0–3 cases annually in the period 2013–2021) and in the EU/EEA (24–57 cases annually in the period 2013–2021) [12], and it is only occasionally detected in environmental sources [13] and wild animals, like birds [14] and amphibians [15]. *S*. Hvittingfoss was first described in 1936 as a causative agent of acute gastroenteritis in Hvittingfoss. Seven people from the same family developed gastroenteritis after eating soft cheese. The new *Salmonella* strain was isolated from stool samples. Infected cows were probably the cause of the cheese contamination [16]. Since then, this serovar has caused a few outbreaks of foodborne infections in Asia, the United States, and Australia. In 1976, *S*. Hvittingfoss was found in stool samples of six Dutch people who returned from a group trip to Japan and Thailand. Five of them had symptoms of food poisoning, and a crab cocktail was pointed out as the possible source of contamination [17]. In 2010, 97 cases of *S*. Hvittingfoss were confirmed among Subway restaurant customers in 28 counties of the state of Illinois. At least 25 people were hospitalized due to a severe illness. The exact food source was never found [18]. In the summer of 2016, an outbreak of *S.* Hvittingfoss occurred across six states of Australia, likely linked to the consumption of rock melons. During the outbreak, 144 cases of *S*. Hvittingfoss were confirmed and two separate strains of *S*. Hvittingfoss were identified via WGS [19]. In 2022, an outbreak was reported in China: 6 out of 18 people of a tourist group who came back from Hong Kong were admitted to the hospital due to severe diarrhea, probably due to the consumption of contaminated beef. *S*. Hvittingfoss was isolated from rectal swabs from these patients [20]. None of these discussed cases of *S*. Hvittingfoss reported symptoms other than gastroenteritis [17,18,19,20]. To the best of our knowledge, bacteremia or focal infections, as well as asymptomatic carriage, were not described for this serovar before.

## 4. Conclusions

In conclusion, we report for the first time an atypical presentation of salmonellosis caused by *S.* Hvittingfoss: a bacteremia and endometritis following a uterine aspiration. As the patient did not show clear symptoms of gastroenteritis, the identification of *Salmonella* as the cause of this infection was rather unexpected. The literature corroborates that *Salmonella* as a cause of urogenital infections is rare but not uncommon. Therefore, identification of *Enterobacterales* in urogenital flora should be considered in cases of severe urogenital infections or a history of invasive urologic procedures, especially when other cultures remain negative. As not all patients develop gastrointestinal symptoms, a link with salmonellosis is not always obvious but might be important as it can be responsible for sepsis or other complications.

## Data Availability

All relevant data are within the manuscript.

## References

[1] Bush L.M., Vazquez-Pertejo M.T. Nontyphoidal Salmonella Infections. https://www.msdmanuals.com/professional/infectious-diseases/gram-negative-bacilli/nontyphoidal-salmonella-infections.

[2] Valayatham V. (2009). Salmonella: The pelvic masquerader. Int. J. Infect. Dis..

[3] Kostiala A., Ranta T. (1989). Pelvic inflammatory disease caused by *Salmonella panama* and its treatment with ciprofloxacin. Case report. Br. J. Obstet. Gynaecol..

[4] Gantois D., Goudard Y., Wolf A., Imbert P., Pauleau G., Sockeel P. (2017). A Rare Case of Genital Salmonella. Ann. Clin. Pathol..

[5] Myrianthefs P., Varvaresou K., Ladakis C., Pactitis S., Lappas V., Stamatiou J., Carousou A., Pavlides J., Baltopoulos J. (2000). Bacteremia and systemic inflammatory response syndrome (SIRS) in confirmed post-partum endometritis. Crit. Care.

[6] Gal-Mor O. (2019). Persistent Infection and Long-Term Carriage of Typhoidal and Nontyphoidal Salmonellae. Clin. Microbiol. Rev..

[7] Sirinivan S., Pokawattana L., Bangtrakulnondh A. (2004). Duration of Nontyphoidal Salmonella Carriage in Asymptomatic Adults. Clin. Infect. Dis..

[8] Leber A.L. (2016). Chapter 3.9.1: Guidelines for Performance of Genital Culture. Clinical Microbiology Procedures Handbook.

[9] Coughlin L.B., McGuigan J., Haddad N.G., Mannion P. (2003). Salmonella sepsis and miscarriage. Clin. Microbiol. Infect..

[10] Zettell L.A., Jelsema R.D., Isada N.B. (1995). First-Trimester Septic Abortion Due to Salmonella enteritidis oranienburg. Infect. Dis. Obstet. Gynecol..

[11] Mollo B., Hobson C.A., Le Hello S., Azria E., Le Monnier A., Pilmis B., Mizrahi A. (2021). Intrauterine infection caused by nontyphoidal Salmonella: A literature review. J. Matern. Neonatal Med..

[12] European Centre for Disease Prevention and Control (ECDC) Surveillance Atlas of Infectious Diseases. http://atlas.ecdc.europa.eu/public/.

[13] Nguyen D., Awasthi S., Hoang P., Nguyen P., Jayedul H., Hatanaka N., Hinenoya A., Van Dang C., Faruque S., Yamasaki S. (2021). Prevalence, Serovar and Antimicrobial Resistance of Nontyphoidal Salmonella in Vegetable, Fruit, and Water Samples in Ho Chi Minh City, Vietnam. Foodborne Pathog. Dis..

[14] Smith H.G., Bean D.C., Hawkey J., Clarke R.H., Loyn R., Larkins J., Hassell C., Valcanis M., Pitchers W., Greenhill A.R. (2020). Salmonella enterica Serovar Hvittingfoss in Bar-Tailed Godwits (*Limosa lapponica*) from Roebuck Bay, Northwestern Australia. Appl. Environ. Microbiol..

[15] Ribas A., Poonlaphdecha S. (2016). Wild-Caught and Farm-Reared Amphibians are Important Reservoirs of Salmonella, A Study in North-East Thailand. Zoonoses Public Health.

[16] Tesdal M. (1936). Ein neuer Salmonellatypus (*Salmonella hvittingfoss*). Z. Für Hyg..

[17] Bijkerk H., Van Os M., Siem T. (1977). Een explosie van voedselvergiftiging onder een groep reizigers. Ned. Tijdschr. Geneeskd..

[18] Greenhalgh M. (2010). Salmonella Lawsuit Filed against Subway. Food Safety News.

[19] Todd K., Beazley R., Furlong C., Kenny B., Shadbolt C., Centofanti A., Schobben X., Polkinghore B., Gregory J., Easton M. A Multi-state Outbreak of *Salmonella hvittingfoss* Associated with Melons in Australia, 2016. Proceedings of the 9th TEPHINET Global Scientific Conference.

[20] Fang Y., Huang H., Li B., Ruan F., Li Z., Huang W., Wei Q., Huang K. (2022). An outbreak of *Salmonella hvittingfoss* infection in a tourist group back from Hong Kong, Southeast China. J. Infect..

